# Supplementary material for: Solvation-property relationship of lithium-sulphur battery electrolytes
Source: Nat Commun. 2024 Feb 10;15:1268. doi: 10.1038/s41467-023-44527-x (PMC10858963; doi:10.1038/s41467-023-44527-x)
Supplement: Supplementary file 1 — Supplementary Information [file 41467_2023_44527_MOESM1_ESM.pdf]

# Supplementary Information

## Solvation-Property Relationship of Lithium-Sulphur Battery Electrolytes

Sang Cheol Kim[1‡], Xin Gao[1‡], Sheng-Lun Liao[2], Hance Su[1], Yuelang Chen[3], Wenbo Zhang[1], Louisa C. Greenburg[1], Jou-An Pan[1], Xueli Zheng[1], Yusheng Ye[1], Mun Sek Kim[2], Philaphon Sayavong[3], Aaron Brest[1], Jian Qin[2*], Zhenan Bao[2*], Yi Cui[1,4,5*]

[1]Department of Materials Science and Engineering, Stanford University, Stanford, California 94305, USA.

[2]Department of Chemical Engineering, Stanford University, Stanford, California 94305, USA.

[3]Department of Chemistry, Stanford University, Stanford, California 94305, USA.

[4]Department of Energy Science and Engineering, Stanford University, Stanford, California 94305, USA.

[5]Stanford Institute for Materials and Energy Sciences, SLAC National Accelerator Laboratory, 2575 Sand Hill Road, Menlo Park, California 94025, USA.

[‡] These authors contributed equally

[*] These authors jointly supervised this work

Correspondence to: Y. C., yicui@stanford.edu, Z. B., zbao@stanford.edu, J. Q., jianq@stanford.edu

**Supplementary Figures**

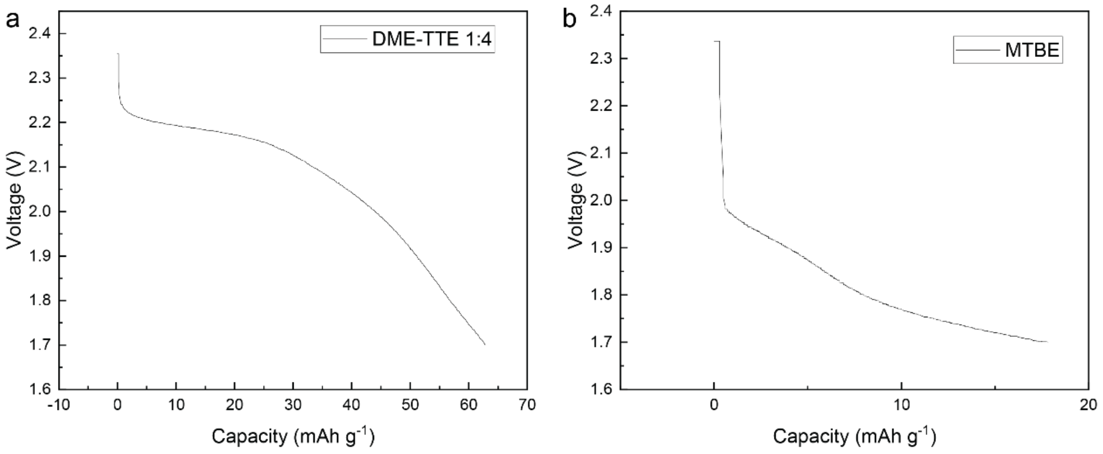


Supplementary Fig. 1 | Discharge capacity of weakly solvating electrolytes, showing that excessively weak solvation will deliver extremely small capacities due to sluggish kinetics. a, DME-TTE 1:4. b, MTBE.

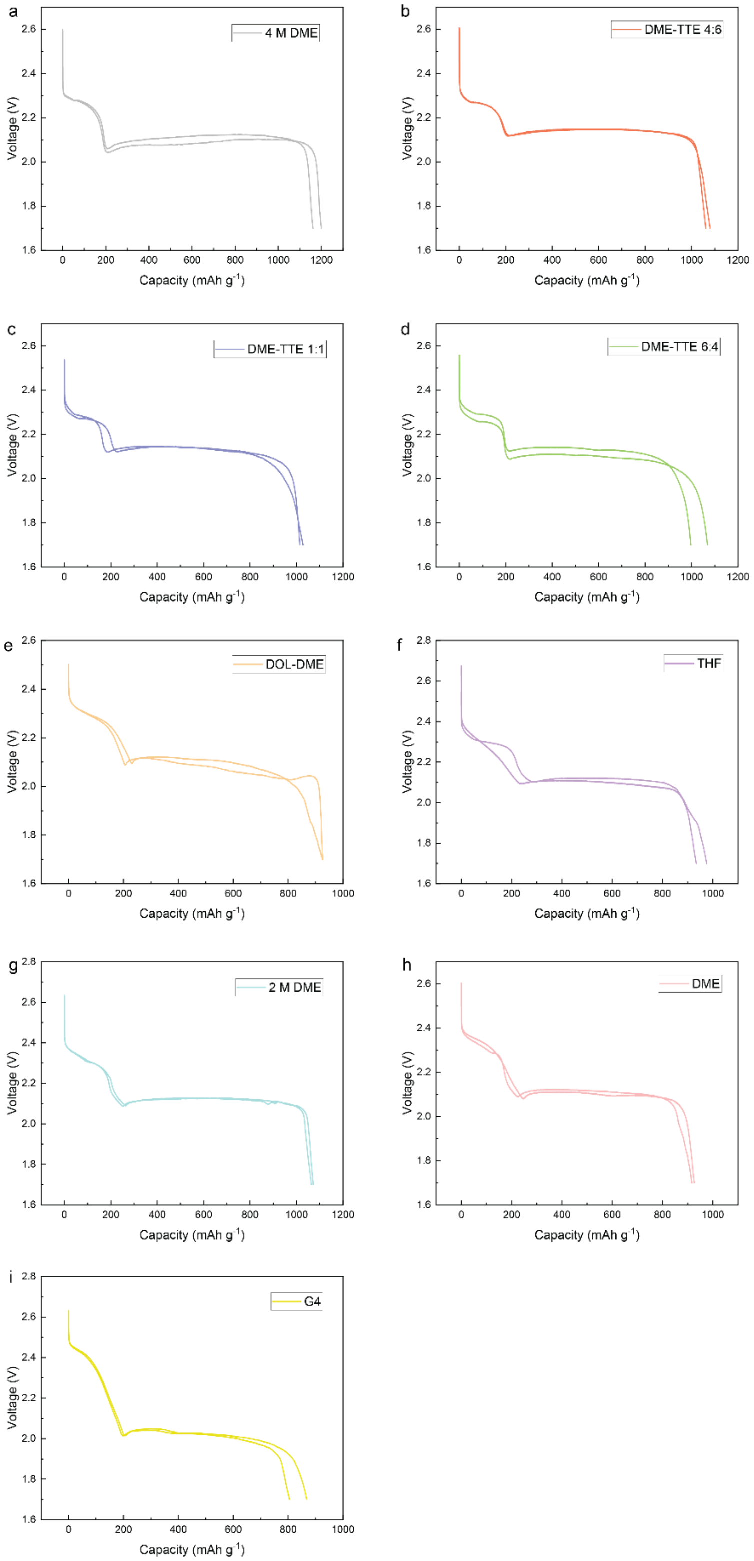


Supplementary Fig. 2 | Discharge voltage profiles of different electrolytes at C/20. a, 4 M DME. b, DME-TTE 4:6. c, DME-TTE 1:1. d, DME-TTE 6:4. e, DOL-DME. f, THF. g, 2 M DME. h, DME. i, G4.

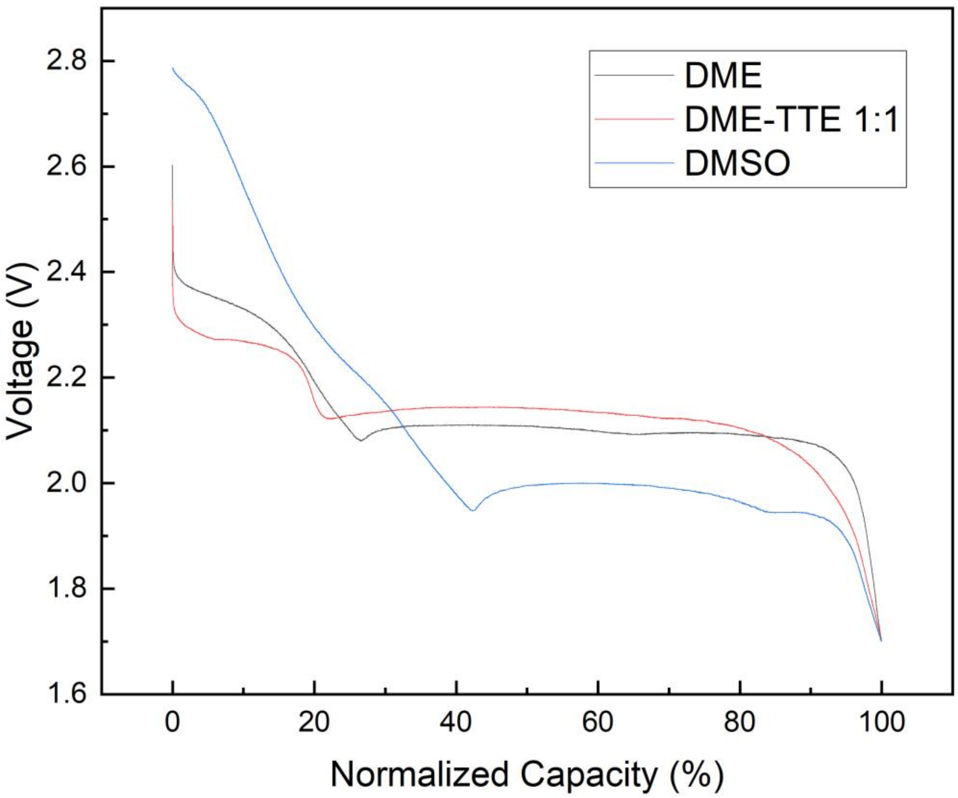


Supplementary Fig. 3 | Voltage profiles of 1 M LiTFSI in DME, DME-TTE 1:1 and DMSO.

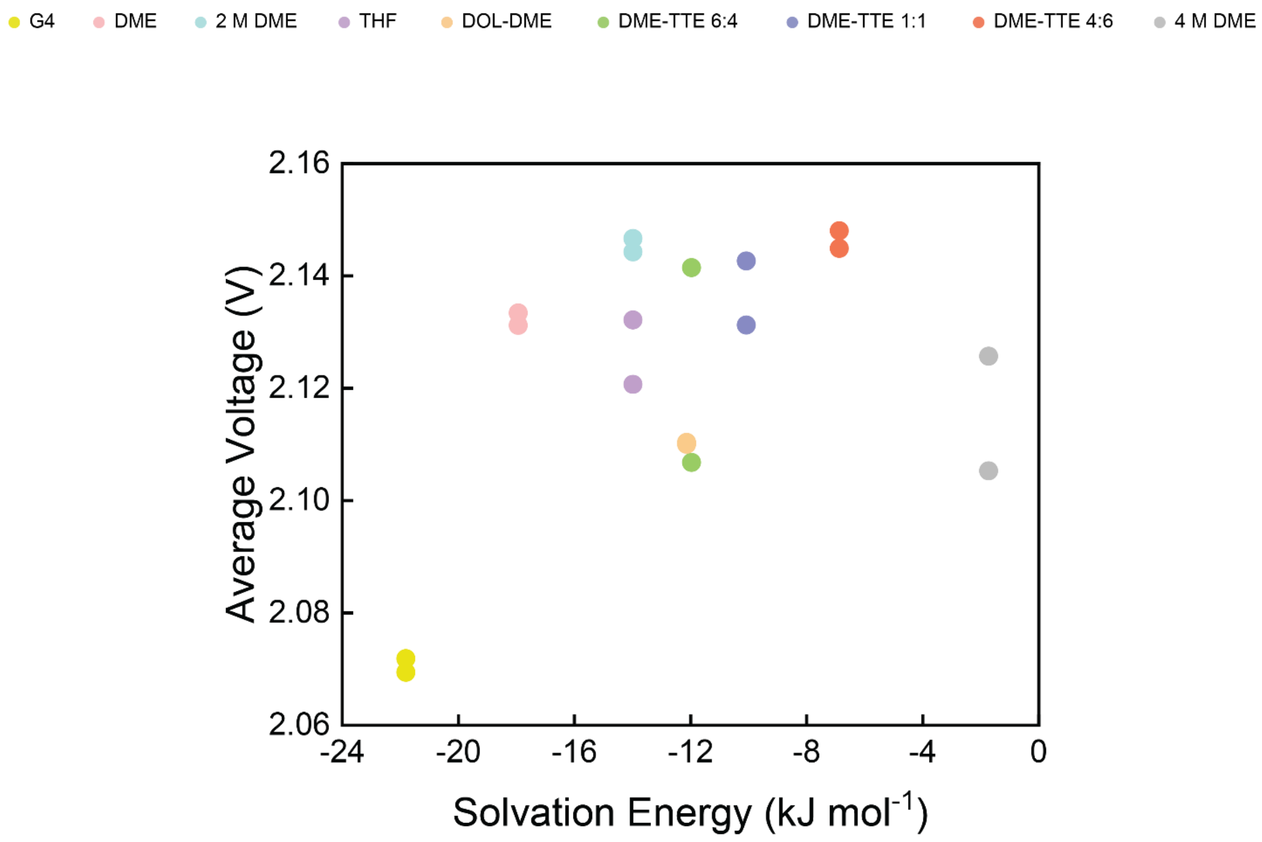


Supplementary Fig. 4 | Correlation between solvation energy and the average discharge voltage, showing a weak positive correlation.

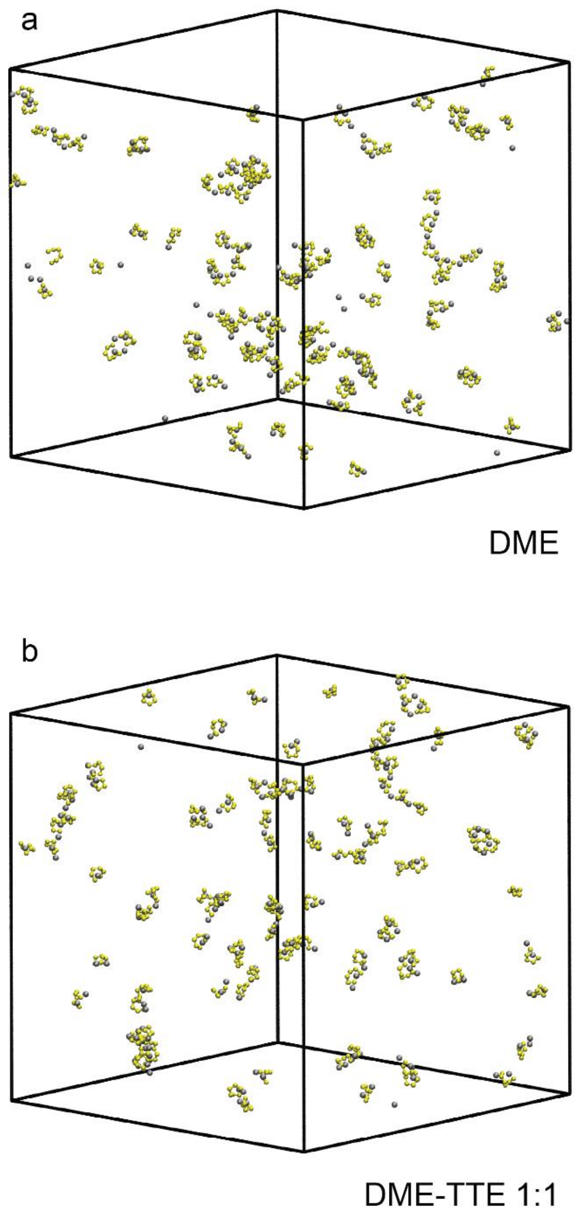


Supplementary Fig. 5 | Snapshots from classical MD simulation for a, 0.1 M $Li_2S_6$ in DME and b, DME-TTE 1:1. Lithium and sulfide are shown as silver and yellow particles, respectively. DME and TTE are omitted from the figures for transparency purposes.

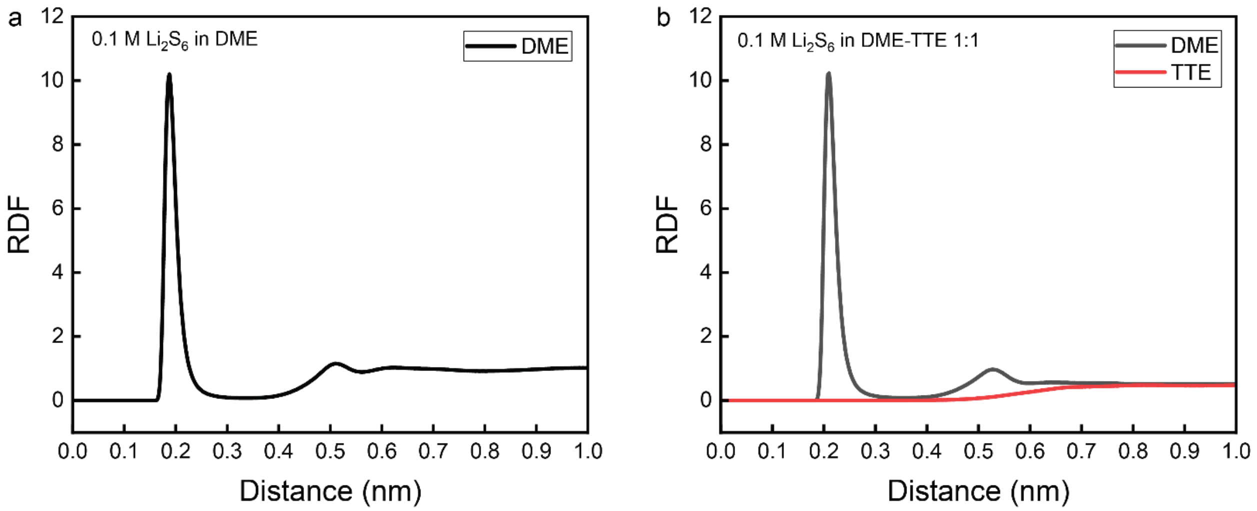


Supplementary Fig. 6 | Radial distribution functions (RDFs) of $Li^+$ in a, 0.1 M $Li_2S_6$ in DME and b, 0.1 M $Li_2S_6$ in DME-TTE 1:1.

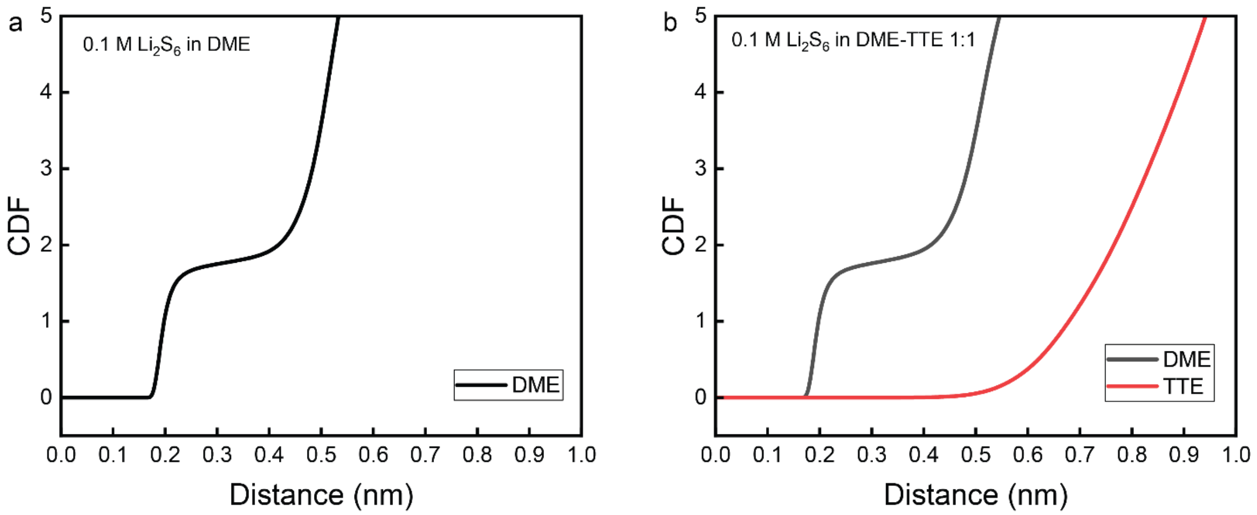


Supplementary Fig. 7 | Cumulative distribution functions (CDFs) of $Li^+$ in a, 0.1 M $Li_2S_6$ in DME and b, 0.1 M $Li_2S_6$ in DME-TTE 1:1.

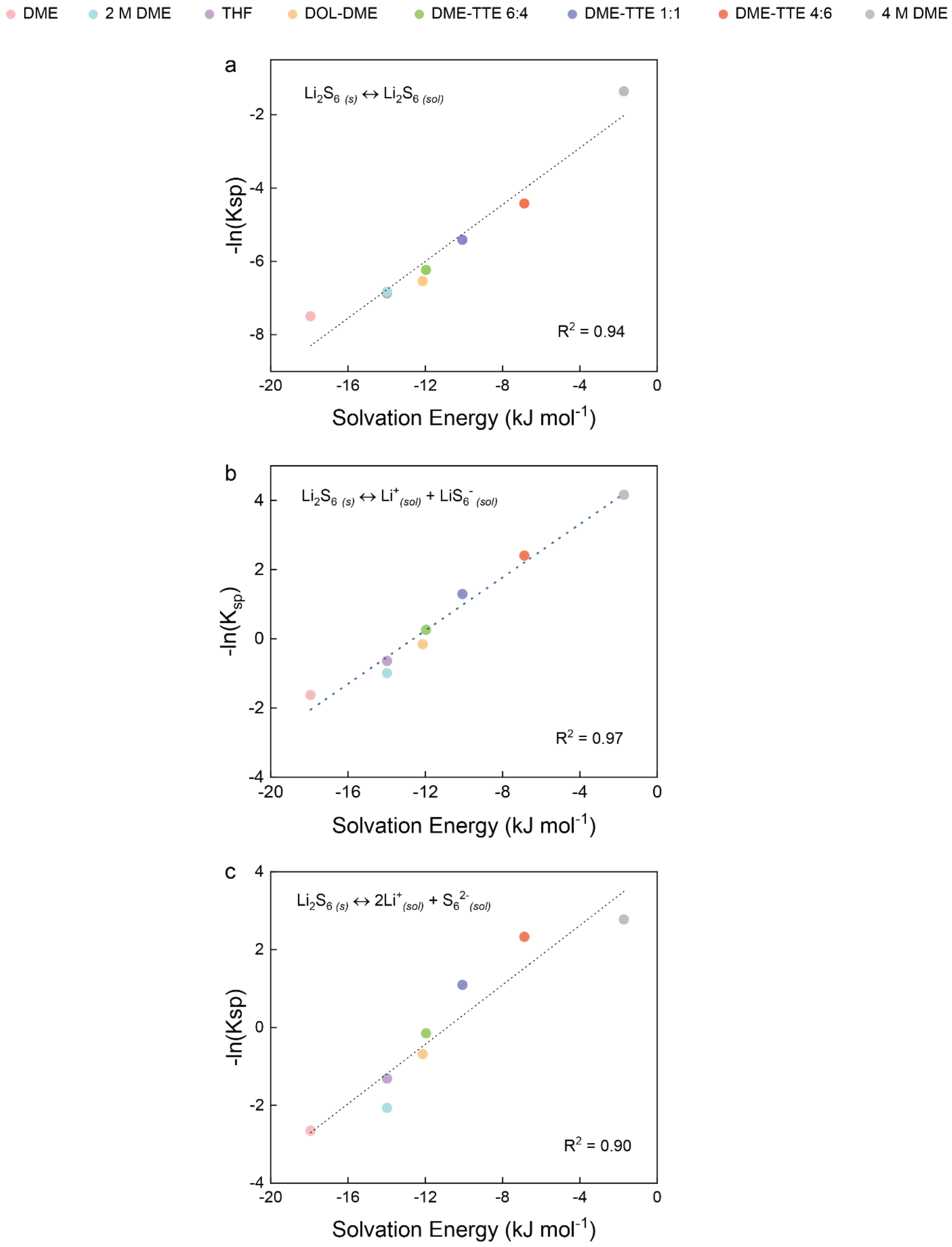


Supplementary Fig. 8 | The relationship between -ln($K_{sp}$) and solvation energy for three different possible dissolution reactions. a, $Li_2S_6$ *(s)* ↔ $Li_2S_6$ *(sol)*. b, $Li_2S_6$ *(s)* ↔ $Li^+$ *(sol)* + $LiS_6^-$ *(sol)*. c, $Li_2S_6$ *(s)* ↔ 2$Li^+$ *(sol)* + $S_6^{`2-}$ *(sol)*.

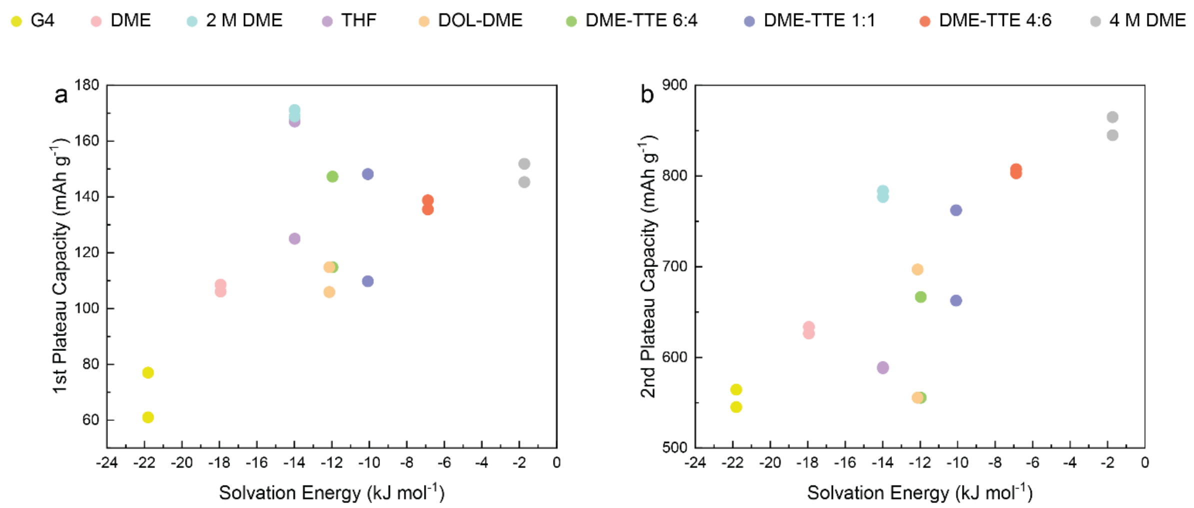


Supplementary Fig. 9 | Correlation between solvation energy and a, the 1st plateau capacity and b, 2nd plateau capacities, showing an increase with weaker solvation.

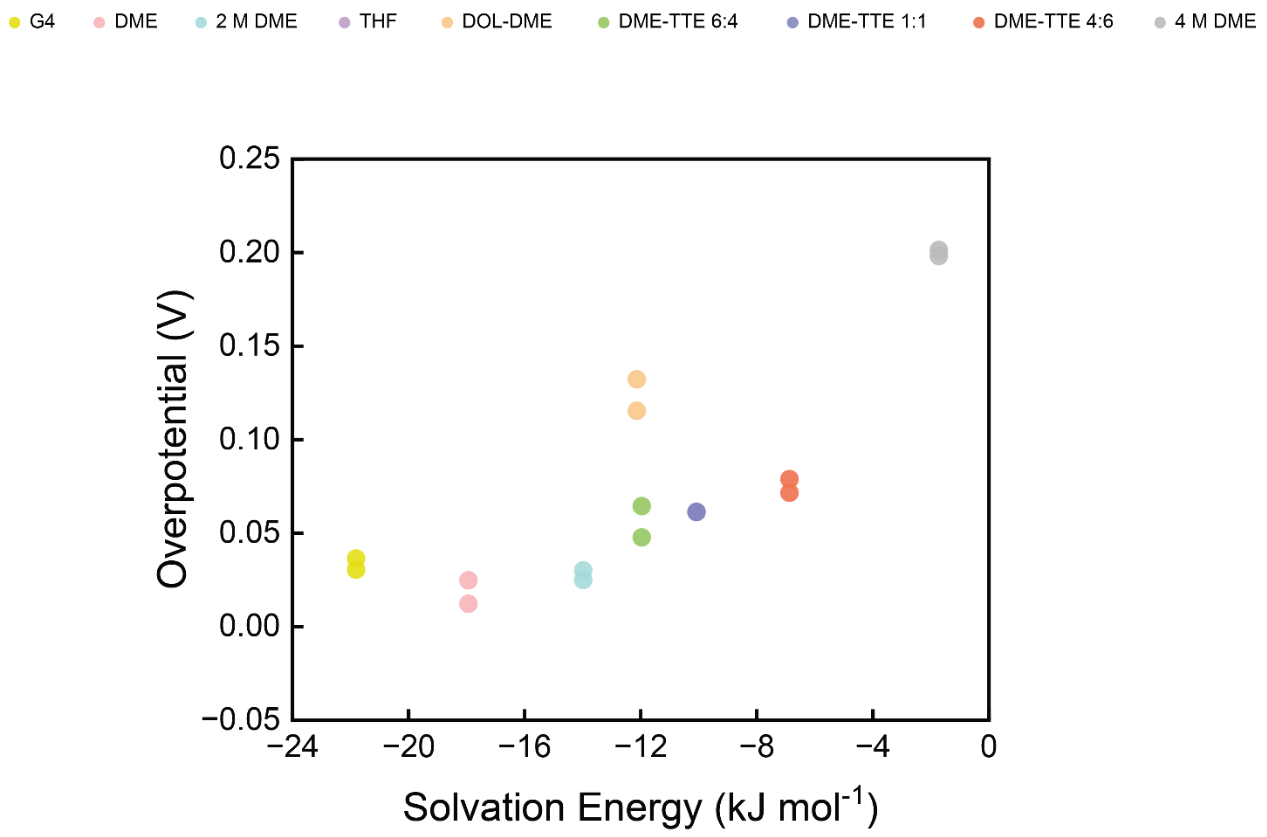


Supplementary Fig. 10 | Correlation of 2nd plateau overpotential, obtained by the plateau voltage differences in 0.2C and 0.05C, to solvation energy.

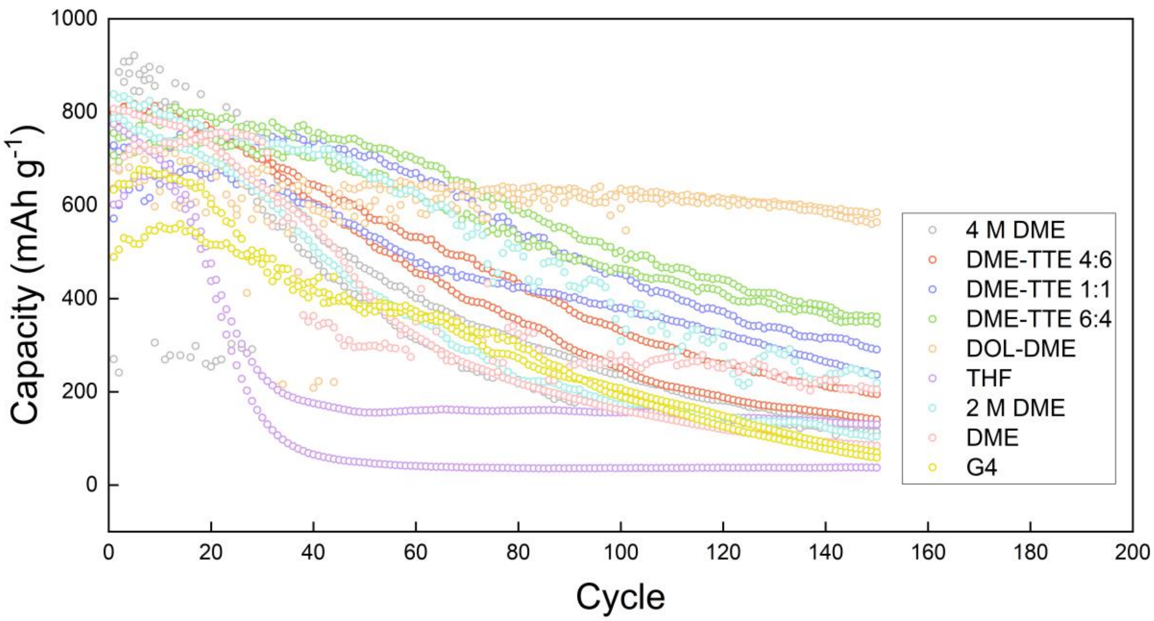


Supplementary Fig. 11 | Li-S battery cycling performance of different electrolytes, cycled at 0.2C.

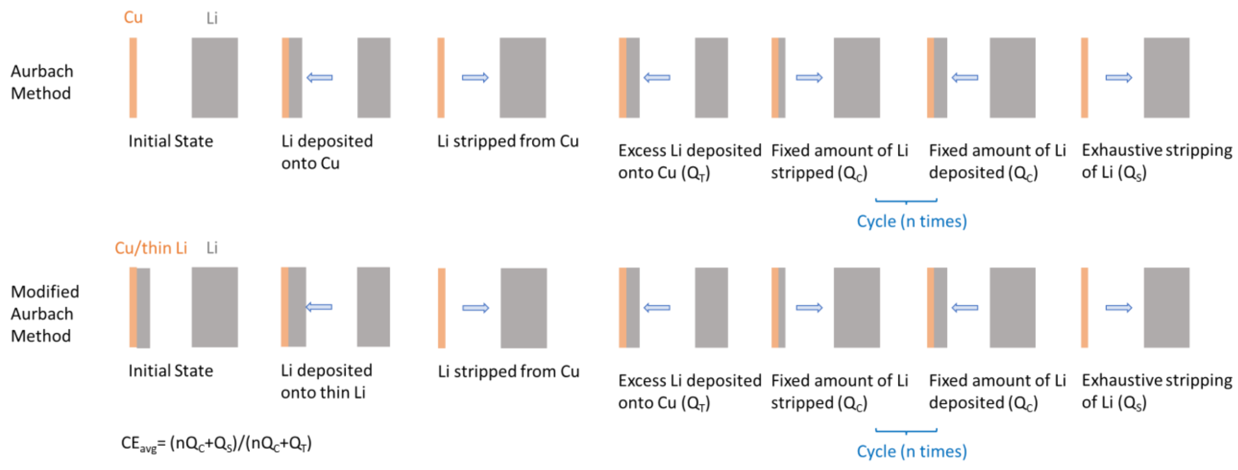


Supplementary Fig. 12 | Schematic outlining the Aurbach method and the modified Aurbach method to measure CE.

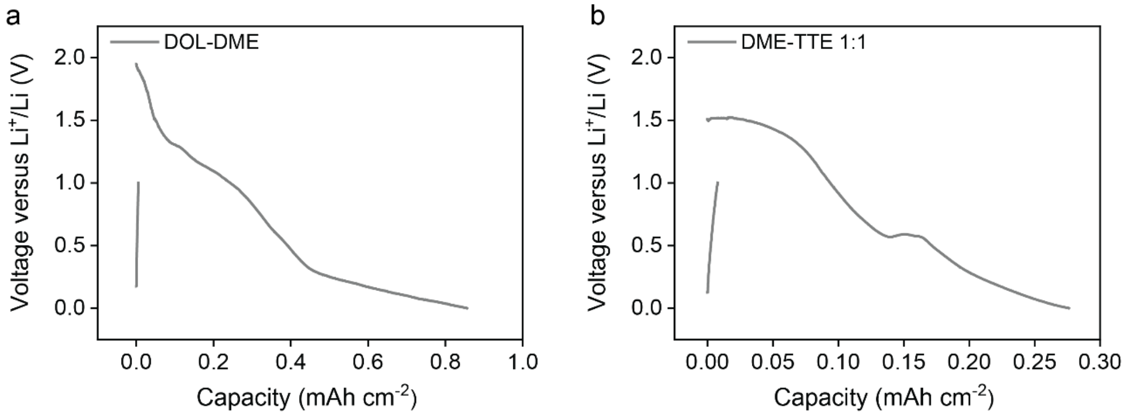


Supplementary Fig. 13 | First discharge curve of a, DOL-DME and b, DME-TTE 1:1, showing the LiPS reduction reactions that hinder accurate measurement of CE.

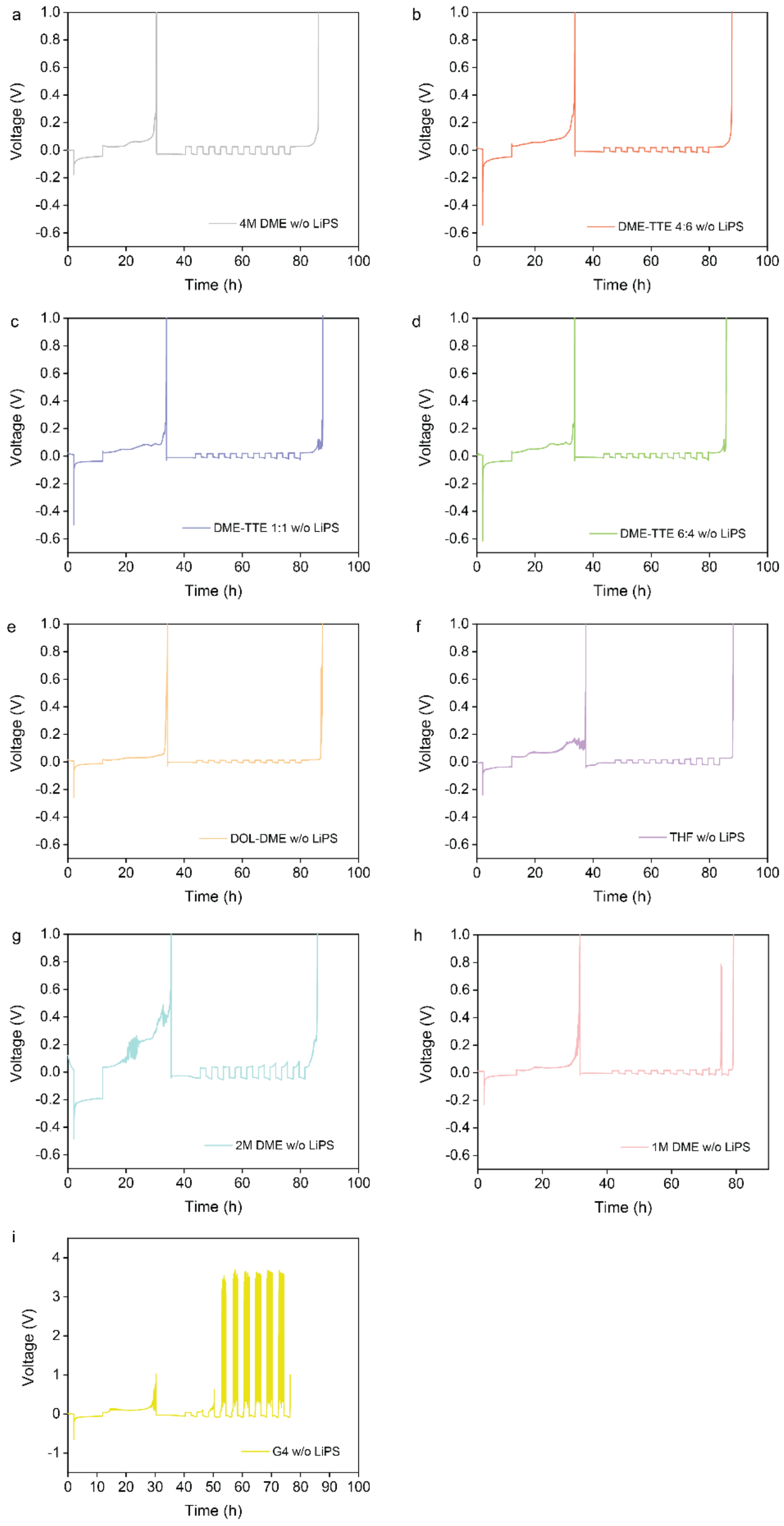


Supplementary Fig. 14 | Voltage profiles of the modified Aurbach method for the electrolyte without LiPS. a, 4 M DME. b, DME-TTE 4:6. c, DME-TTE 1:1. d, DME-TTE 6:4. e, DOL-DME. f, THF. g, 2 M DME. h, DME. i, G4.

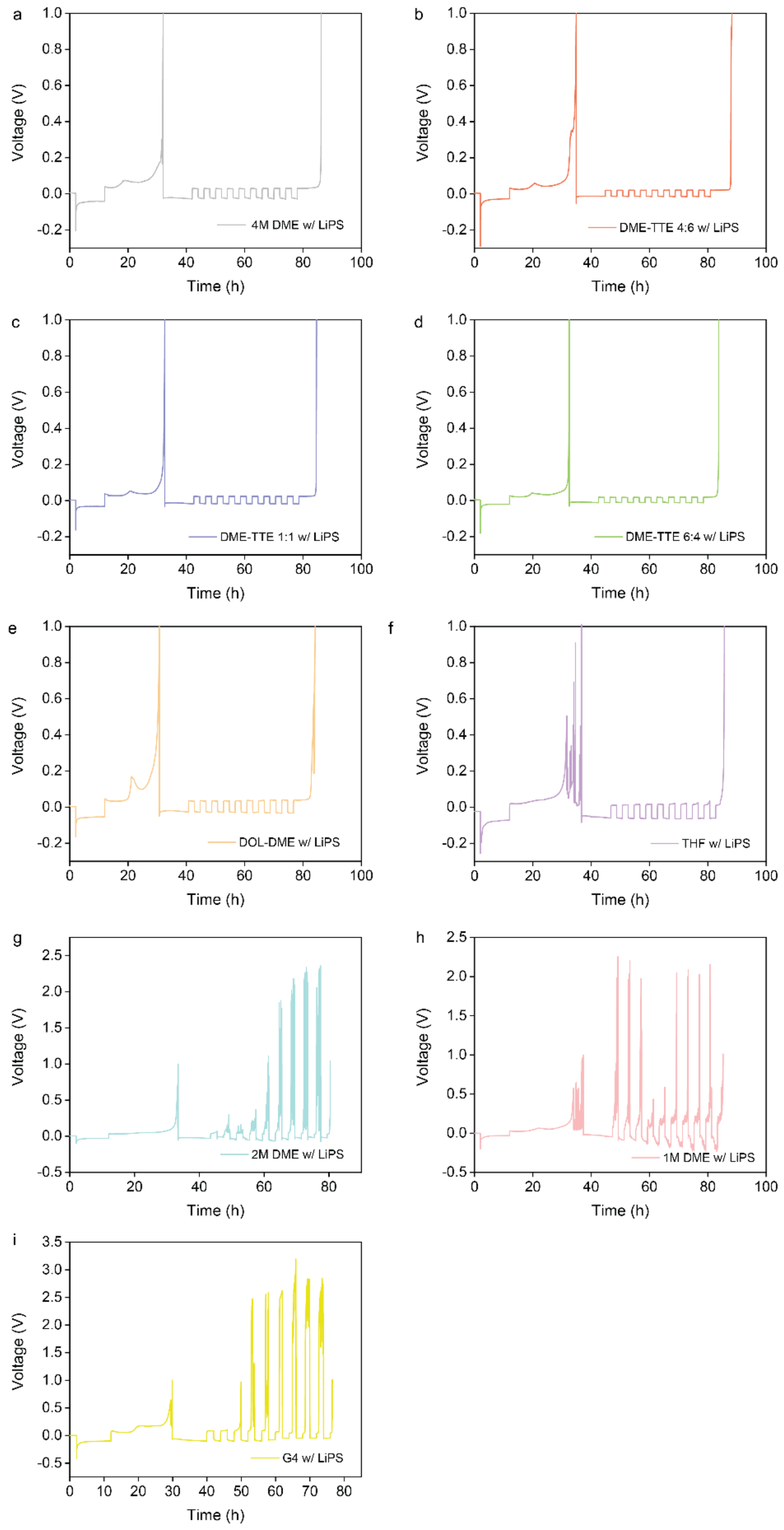


Supplementary Fig. 15 | Voltage profiles of the modified Aurbach method for the electrolyte with LiPS. a, 4 M DME. b, DME-TTE 4:6. c, DME-TTE 1:1. d, DME-TTE 6:4. e, DOL-DME. f, THF. g, 2 M DME. h, DME. i, G4.
